# HLA‐DQB1*05:02: A Novel Genetic Marker for Susceptibility to Oral Cavity Squamous Cell Carcinoma

**DOI:** 10.1002/cam4.70876

**Published:** 2025-04-15

**Authors:** Shiang‐Fu Huang, Huei‐Tzu Chien, Chi‐Kuang Young, Yun‐Shien Lee, Chun‐Ta Liao, Kai‐Lun Cho, Ching‐Han Chen, Angel Chao

**Affiliations:** ^1^ Department of Otolaryngology, Head and Neck Surgery Chang Gung Memorial Linkou Taiwan; ^2^ Graduate Institute of Clinical Medical Sciences Chang Gung University Taoyuan Taiwan; ^3^ Department of Nutrition and Health Sciences Chang Gung University of Science and Technology Taoyuan Taiwan; ^4^ Research Center for Chinese Herbal Medicine, College of Human Ecology Chang Gung University of Science and Technology Taoyuan Taiwan; ^5^ Department of Otolaryngology, Head and Neck Surgery Chang Gung Memorial, Keelung Branch Keelung Taiwan; ^6^ Medical College Chang Gung Memorial Hospital Kwei‐Shan Taoyuan Taiwan; ^7^ Department of Biotechnology Ming Chuan University Taoyuan Taiwan; ^8^ School of Medicine, Chang Gung Medical College Chang Gung University Taoyuan Taiwan; ^9^ Department of Obstetrics and Gynecology Chang Gung Memorial Hospital and Chang Gung University Taoyuan Taiwan

**Keywords:** genome‐wide association study, HLA‐DQB1, oral squamous cell carcinoma, susceptibility, young age

## Abstract

**Introduction:**

Environmental exposure to carcinogens causes mucosal damage in the upper aerodigestive tract and can lead to cancers. The susceptibility to carcinogens varies between individuals. To identify susceptibility genes in head and neck cancers, we enrolled patients with early‐onset disease and analyzed them by genome‐wide association study.

**Methods and Materials:**

This case–control study included 54 young male patients with oral squamous cell carcinoma (OSCC) who were treated between March 1996 and December 2016, as well as 2400 healthy controls. A single nucleotide polymorphism (SNP) array was used to determine genetic loci that increase susceptibility to OSCC. In another validation cohort, sequencing‐based typing (TBG Biotechnology Corp., Taipei, Taiwan) was used to determine the HLA‐DQB1 genotype in another 100 OSCC patients.

**Results:**

We analyzed the allele frequencies of 664,994 autosomal SNPs in 54 OSCC cases. In a genome‐wide association analysis, four SNPs on chromosomes 6, 7, 9, and 12 were significantly different between OSCC patients and controls (corrected *p* < 1.0 × 10^−6^). HLA‐DQB1 was closest to rs28451423 on chromosome 6. In the validation cohort, HLA‐DQB1*05:02 in OSCC (18.5%) was significantly different from the normal population (7.0%) (*p* = 0.009). The influence of disease onset was independently significant after adjusting smoking, alcohol drinking, and areca‐quid chewing (*p* = 0.015, OR: 3.922, 95% confidence interval: 1.311–11.734). Furthermore, HLA‐DQB1*05:02 was associated with early‐onset OSCC (*p* = 0.004).

**Conclusion:**

HLA‐DQB1*05:02 increases individuals' risks of OSCC independent of environmental exposures, particularly in cases of early‐onset OSCC. This study provides a genetic basis and disease marker for personalized prevention of OSCC.

## 1 Introduction

In Taiwan, oral cavity squamous cell carcinoma (OSCC) is the third most common cancer in males [1]. After correcting for age, the incidence of OSCC is much higher in males than females, with a male to female ratio of 14:1 [2]. This discrepancy might be due to differences in the rates of cigarette smoking, betel quid chewing, and alcohol consumption between genders [3]. Cigarette, areca quid (AQ), and alcohol are the main environmental carcinogens contributing to OSCC in Southeast Asian countries, including Taiwan.

From the Taiwan National Health Insurance Database between 1997 and 2013, a total of 55,916 patients with head and neck cancer (HNC) had affected first‐degree relatives. A familial aggregation phenomenon was observed among these patients (odds ratio [OR] = 2.04) [4]. In addition, HNC patients with familial aggregation tended to manifest at a younger age compared with sporadic cases. Genetic factors as well as environmental factors played important roles in the development of HNC.

Regarding cancer susceptibility, patients with early‐onset HNC tend to have cigarette smoking, AQ chewing, and alcohol drinking. But the genetic alterations predispose them to malignancies at a younger age were rarely investigated in the literature. Genetic polymorphisms of alcohol‐metabolizing enzymes (*ALDH2* and *ADH1B*) increase the risk of developing aerodigestive tract [5], oropharyngeal, and hypopharyngeal cancers [6], suggesting the influence of genetics on the development of HNC. Within the TCGA database, tongue cancer specimens from young and old patients displayed similar transcriptomic and genomic features, with no significant differences in cancer driver genes, germline predisposition genes, or the burden of somatic single nucleotide variations. Copy number variations were found to occur more frequently in young patients and are correlated with distinct clinical outcomes [7].

In the present study, we investigated susceptibility genes in OSCCs from early‐onset patients that could harbor specific genetic variations associated with OSCCs by using a high‐throughput genomic approach.

## 2 Materials and Methods

We investigated the susceptibility genes associated with early‐onset (≤ 40 years of age) OSCC using a single nucleotide polymorphism (SNP) array. The study design followed the Strengthening the Reporting of Observational Studies in Epidemiology (STROBE) guidelines [8] and the checklist was in the Supporting Information of this article.

### 2.1 Participants

We recruited two cohorts: one is the discovery cohort for the SNP array analysis (with 54 young age OSCC patients and 2400 normal population) and second is the validation cohort for the HLA typing to investigate the locus identified in the discovery cohort (100 OSCC patients and 50 normal population that are completely independent from the discovery cohort).

#### 2.1.1 Discovery Cohort

The diagnosis of OSCC was confirmed histologically in all patients (*n* = 54). Prior to surgery, all patients provided written informed consent for participation in a questionnaire survey that included items related to demographic factors, gender, family history, smoking, alcohol consumption, and habitual use of AQ. Patients with a diagnosis other than OSCC were excluded. Age, tumor stage, and tumor subsites were determined by reviewing electronic medical records. The study protocol was approved by the Institutional Review Board of Chang Gung Medical Foundation (201800439B0). Additionally, we recruited 2400 ethnically and geographically matched healthy controls from a biobank for a nationwide population study (TMU‐201805076) [9]. Informed consent was obtained from all participants. The controls were recruited from Taiwan, where 98% of the population is Han Chinese, with a minor Hakka Chinese population [10].

#### 2.1.2 Validation Cohort

All patients in this cohort were separately recruited and independent from the discovery cohort. All the OSCC patients (*n* = 100) were confirmed histologically. All patients provided written informed consent for participation in a questionnaire survey as the discovery cohort. The clinicopathological data were recorded as described in the discovery cohort. Normal controls in this cohort were recruited from patients who have undergone surgeries for chronic tonsillitis and chronic rhinitis. All the normal controls were proven to not have malignancies.

### 2.2 DNA Extraction

In all participants, 10‐mL venous blood was collected in a vacuum tube containing an anticoagulant (Vacutainer; BD Biosciences, Franklin Lakes, NJ, USA). The buffy coat obtained from this sample was isolated and stored at −80°C. High‐molecular‐weight DNA was extracted from buffy coat cells using the standard phenol‐chloroform method and then stored at −80°C [10]. To evaluate DNA quantity and purity, spectrophotometry was performed using the BioDrop μLite spectrophotometer (BioDrop, Cambridge, UK). An optimal absorbance ratio of 260/280 and a purity index of > 1.8 were considered optimal. For array analysis, each sample had a volume of 50 μL and a concentration of 15 ng/μL.

### 2.3 SNP Array Genotyping and Quality Control in Discovery Cohort

A genome‐wide association study (GWAS) of all samples, featuring 703,949 SNPs, obtained from 54 early‐onset HNC patients and 2400 controls, was performed using the Axiom Genome‐Wide TWB Array Plate (Affymetrix GeneTitan; Thermo Fisher Scientific, Waltham, MA, USA) [11]. Multidimensional scaling analysis was applied to assess the genome‐wide identity, and outliers deviating from clusters were eliminated. We excluded markers that did not meet the following thresholds: missing rate < 2%, minor allele frequency rate > 5%, and Hardy–Weinberg equilibrium *p* > 0.0001. The replication samples underwent the same quality control measures.

### 2.4 HLA‐DQB1 Genotyping in Validation Cohort

HLA‐DQB1 alleles were identified using the HLAssure SE DQB1 Locus SBT Kit (Catalog no. 50510; TBG Biotechnology Corp., Taipei, Taiwan). This kit is used to perform direct sequence‐based typing based on capillary sequencing, which is widely recognized as the gold standard for HLA typing [12]. This method can identify the known polymorphic positions in Exons 2 and 3 of HLA‐DQB1, which are crucial for precise allelic determination. Our HLA‐typing experiment was conducted in accordance with the manufacturer's instructions. The sequencing results were processed using the provided licensed software (AccuType Software), following the manufacturer's instructions. The HLA‐DQB1 genotypes were compared among the groups.

### 2.5 Statistical Analysis in the Validation Cohort

The frequencies of HLA‐DQB1 alleles were determined by direct counting. To compare the allele frequencies of HLA‐DQB1 between the young and old OSCC groups in the validation cohort, the chi‐square test or Fisher's exact test was used, as appropriate. Bonferroni correction was applied by multiplying *p* values to calculate corrected *p* values. ORs with 95% confidence intervals (CIs) were used to quantify the effect of DQB1 on the susceptibility to early‐onset OSCC. The age distribution of OSCC patients according to the HLA‐DQB1 genotype was visualized using a histogram and compared using the independent *t*‐test. Correlation plots were generated using the corrplot package (version 0.92; R Foundation for Statistical Computing, Vienna, Austria). Overall survival and disease‐free survival in the validation cohort were assessed using the Kaplan–Meier method and compared among groups using the log‐rank test. Statistical analyses were performed using R software (version 4.2.2) [13].

## 3 Results

### 3.1 SNP Array Analysis in Discovery Cohort

In the discovery cohort, we recruited a total of 54 OSCC patients and 2400 controls (Table 1). The GWAS was conducted for 54 OSCC patients and 2387 controls due to some patients' DNA quality was not suitable for experiments (*n* = 13). The TW2.0 SNP chip was used to analyze 703,949 variants. Following quality control procedures and the removal of variants with a minor allele frequency < 1%, 468,640 variants and 2441 samples (including 54 cases and 2387 controls) were analyzed. SNPs on the sex chromosomes were excluded from the GWAS. Table 1 presents the demographic data of the participants in the discovery cohort. The OSCC population was narrowed to include only young individuals (aged ≤ 40 years). Notably, cigarette smoking, alcohol consumption and AQ consumption were significantly more common in OSCC patients than in controls (*p* < 0.001).

**TABLE 1:** Demographic data of young age OSCC.

	Discovery cohort		Validation cohort	
	Cancer (*n* = 54)	Normal (*n* = 2400)	Cancer (*n* = 100)	Normal (*N* = 50)
Age				
Mean (±SD)	34.91 (±3.78)	48.2 (±8.31)	44.6 (±12.37)	50.2 (±11.27)
Range	25–40		26–70	
Cancer site				
Tongue	25 (46.3)		38 (38.0)	
Lip	1 (1.85)		4 (4.0)	
Buccal mucosa	19 (35.19)		39 (39.0)	
Alveolus	4 (7.41)		9 (9.0)	
Hard palate	2 (3.70)		4 (4.0)	
Retromolar trigone	3 (5.56)		6 (6.0)	
Cigarette				
Yes	50 (92.6)	1100 (45.8)	94 (94.0)	18 (36.0)
No	4 (7.4)	1300 (54.2)	6 (6.0)	32 (64.0)
Alcohol				
Yes	41 (75.9)	428 (17.8)	86 (86.0)	16 (32.0)
No	13 (24.1)	1972 (82.2)	14 (14.0)	34 (68.0)
Areca quid				
Yes	51 (94.4)	411 (17.1)	93 (93.0)	8 (16.0)
No	3 (5.6)	1988 (82.8)	7 (7.0)	42 (84.0)
Stage				
I	9 (16.67)		22 (22.0)	
II	18 (33.33)		26 (26.0)	
III	7 (12.96)		14 (14.0)	
IV	20 (37.04)		38 (38.0)	

**FIGURE 1:**
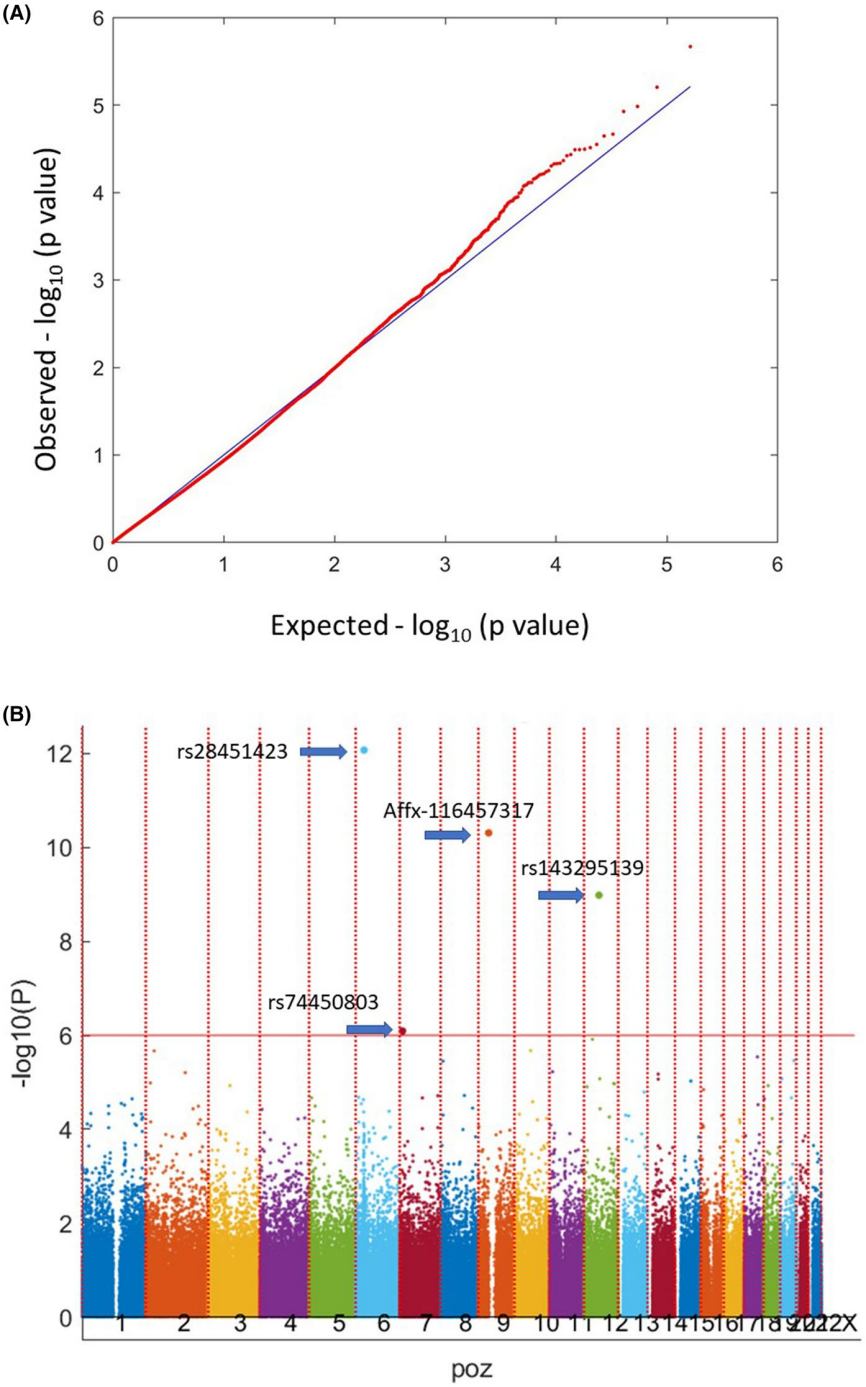
(A) Quantile–quantile plot of the test statistics in case–control study for young age OSCC. The theoretical quantile–quantile plot of the *χ* ^2^ statistics in the case–control study. The black line represents the null model, in which the observed *χ* ^2^ values match the expected values. The red dots represent the observed *χ* ^2^ values compared with the expected values from the case–control study. The genomic inflation factor was 1.061, indicating an acceptable population structure in the GWAS. (B) Manhattan plot of the genome‐wide association results. The *y*‐axis corresponds to −log_10_ *p* values and the *x*‐axis corresponds to the genomic positions. The horizontal red line (*p* = 1 × 10^−6^) denotes the false discovery rate. The four specific SNPs that were statistically significant were labeled as arrows.

The principal component analysis plot demonstrated no significant differences in ancestry distribution between OSCC patients and controls. A quantile–quantile plot was employed for quality control, and it demonstrated successful population matching. The genomic inflation factor was 1.061, indicating an acceptable population structure in the GWAS (Figure 1A). The false discovery rate was used to determine a cut‐off *p* value of 1 × 10^−6^. In the analysis of all early‐onset HNC patients (*n* = 54), using the hg38 reference sequence (https://genome.ucsc.edu) and adjusting for environmental exposures, including cigarette smoking, alcohol consumption, and AQ consumption, early‐onset OSCC was strongly correlated with rs28451423, rs74450803, Affx‐116457317, and rs143295139 (https://genome.ucsc.edu; hg38) (Figure 1B and Table 2). The nearby candidate genes were HLA‐DQB1, PHF14, ANKRD20A2P, and LOC105369787, with minor allele frequencies of 35.64%, 4.62%, 15.87%, and 5.38%, respectively. The minor allele frequencies of PHF14 and LOC105369787 were low, and ANKRD20A2P was identified as a pseudogene. Therefore, we focused on HLA‐DQB1 for further analysis.

**TABLE 2:** The data of four candidate genes that were significantly different in young age OSCC in the discovery cohort from the genome‐wide association analysis.

	rs28451423	rs74450803	Affx‐116457317	rs143295139
Chromosome	6	7	9	12
RS#	rs28451423	rs74450803	Affx‐116457317	rs143295139
Position	32659415	11028274	40243677	58297689
Minor allele	T	G	C	C
*p*	8.44216e‐13	8.10075e‐07	4.88707e‐11	1.03866e‐09
Odds ratio	5.73973	5.85850	1530.24000	14.56840
95% CI (lower)	3.55610	2.90220	171.91200	6.16322
95% CI (upper)	9.26424	11.82620	13,621.00000	34.43650
Nearby GENE	HLA‐DQB1	PHF14	ANKRD20A2P	LOC105369787
Position 1	32659466	10973871	40222101	58328380
Position 2	32666657	11110321	40266926	58336628
A allele	A	A	G	T
B allele	T	G	C	C
Control_AA	1631	2284	2396	2367
Control_AB	726	112	1	25
Control_BB	1	2	0	0
Control_NA	42	2	3	8
Case_AA	122	177	129	175
Case_AB	7	18	60	19
Case_BB	66	0	0	1
Case_NA	0	0	6	0
Minor allele freq	35.64%	4.62%	15.87%	5.38%

### 3.2 HLA‐DQB1 Genotyping in Validation Cohort

In the validation cohort, HLA‐DQB1 genotyping was performed for 100 OSCC patients and normal controls (*n* = 50) (Table 1). Table 3 presents the genotype frequencies of the OSCC patients. Considering the sample size and ethnicity, normal control data for HLA‐DQB1 were obtained from 50 patients without any malignancies or autoimmune diseases. Among the OSCC patients, the most prevalent HLA‐DQB1 genotype was DQB1*03:01 (27.00%), followed by DQB1*05:02 (18.50%) and DQB1*03:03 (15.50%). Notably, HLA‐DQB1*05:02 was significantly more common in OSCC patients (18.50%) than in controls (7.0%; Fischer's exact test, *p* = 0.009). HLA‐DQB1*05:02 was significantly more common in young (22.0%) than in old (15.0%) patients. The difference of HLA‐DQB1*05:02 between young age OSCC and normal population is more significant (*p* = 0.004, OR: 3.747, 95% CI: 1.520–9.237).

**TABLE 3:** The frequencies between HLA‐DQB1 genotype in the validation cohort (oral cancer patients [*n* = 100] and normal population [*n* = 50]).

	Cancer patients		Normal population		Fisher *p* value	*χ* ^2^ *p* value	Odds ratio	95% CI (lower)	95% CI (upper)
	With, *n* (%)	Without, *n* (%)	With, *n* (%)	Without, *n* (%)					
All patients (100 samples)									
DQB1*0201	7 (3.5)	193	5 (5.0)	95	0.543	0.532	0.689	0.213	2.228
DQB1*0301	54 (27.0)	146	23 (23.0)	77	0486	0.454	1.238	0.707	2.164
DQB1*0302	9 (4.5)	191	11 (11.0)	89	**0.048**	**0.033**	0.381	0.153	0.953
DQB1*0303	31 (15.5)	169	15 (15.0)	85	1.000	0.910	1.039	0.532	2.030
DQB1*0401	13 (6.5)	187	9 (9.0)	91	0.483	0.434	0.702	0.290	1.705
DQB1*0402	5 (2.5)	195	2 (2.0)	98	1.000	0.787	1.256	0.239	6.592
DQB1*0501	2 (1.0)	198	1 (1.0)	99	1.000	1.000	1.000	0.090	11.162
DQB1*0502	37 (18.5)	163	7 (7.0)	93	**0.009**	0.008	3.016	1.293	7.034
DQB1*0503	6 (3.0)	194	5 (5.0)	95	0.515	0.385	0.588	0.175	1.974
DQB1*0601	24 (12.0)	176	13 (13.0)	87	0.853	0.804	0.913	0.443	1.879
DQB1*0602	5 (2.5)	195	4 (4.0)	96	0.487	0.473	0.615	0.162	2.344
Old‐age patients (50 patients/samples)									
DQB1*0201	3 (3.0)	97	5 (5.0)	95	0.721	0.470	0.588	0.137	2.528
DQB1*0301	29 (14.5)	71	23 (23.0)	77	0.420	0.333	1.367	0.725	2.581
DQB1*0302	7 (7.0)	93	11 (11.0)	89	0.459	0.323	0.609	0.226	1.641
DQB1*0303	11 (11.0)	89	15 (15.0)	85	0.529	0.400	0.700	0.305	1.611
DQB1*0401	9 (9.0)	91	9 (9.0)	91	1.000	1.000	1.000	0.380	2.634
DQB1*0402	3 (3.0)	97	2 (2.0)	98	1.000	0.651	1.515	0.248	9.270
DQB1*0501	2 (2.0)	98	1 (1.0)	99	1.000	0.561	2.020	0.180	22.645
DQB1*0502	15 (15.0)	85	7 (7.0)	93	0.112	0.071	2.344	0.912	6.027
DQB1*0503	1 (1.0)	99	5 (5.0)	95	0.212	0.097	0.192	0.022	1.673
DQB1*0601	14 (14.0)	86	13 (13.0)	87	1.000	0.836	1.089	0.484	2.453
DQB1*0602	3 (3.0)	97	4 (4.0)	96	1.000	0.700	0.742	0.162	3.405
Young‐age patients (50 patients/samples)									
DQB1*0201	4 (4.0)	96	5 (5.0)	95	1.000	0.733	0.792	0.206	3.039
DQB1*0301	25 (25.0)	75	23 (23.0)	77	0.869	0.741	1.116	0.583	2.136
DQB1*0302	2 (2.0)	98	11 (11.0)	89	**0.018**	**0.010**	0.165	0.036	0.765
DQB1*0303	20 (20.0)	80	15 (15.0)	85	0.251	0.151	0.421	0.125	1.416
DQB1*0401	4 (4.0)	96	9 (9.0)	91	0.251	0.152	0.421	0.125	1.416
DQB1*0402	2 (2.0)	98	2 (2.0)	98	1.000	1.000	1.000	0.138	7.242
DQB1*0501	0 (0.0)	100	1 (1.0)	99	1.000	0.316	0	0	NA
DQB1*0502	22 (22.0)	78	7 (7.0)	93	**0.004**	**0.003**	3.747	1.520	9.237
DQB1*0503	5 (5.0)	95	5 (5.0)	95	1.000	1.000	1	0.280	3.567
DQB1*0601	10 (10.0)	90	13 (13.0)	87	0.658	0.506	0.744	0.310	1.785
DQB1*0602	2 (2.0)	98	4 (4.0)	96	0.683	0.407	0.490	0.088	2.737

*Note:* Old age, ≥ 55 years old; Young age, < 40 years old. Bold numbers denote *p* value < 0.05.

The most common causes of OSCC in AQ endemic regions are cigarette smoking, alcohol drinking, and AQ chewing from epidemiologic studies [14]. We further evaluate the role of HLA‐DQB1*05:02 in the onset of OSCC by multivariate analysis adjusting environmental exposures in the validation cohort. In Table 4, binary logistic regression showed AQ chewing has the greatest influence on the occurrence of OSCC (*p* < 0.001, OR: 15.756, 95% CI: 5.344–46.453). The OR of the HLA‐DQB1*05:02 on OSCC is 3.922 (*p* = 0.015, 95% CI: 1.311–11.734). HLA‐DQB1*05:02 is an independent determinant for OSCC.

**TABLE 4:** Multivariate analysis by binary logistic regression of relevant habitual exposures for oral cancer in validation cohort.

		*p*	OR	95% CI
Cigarette smoking	No	**0.048**	Ref	1
	Yes		2.727	1.009–7.372
Alcohol drinking	No	0.262	Ref	1
	Yes		0.559	0.202–1.545
AQ chewing	No	**0.000**	Ref	1
	Yes		15.756	5.344–46.453
HLA DBQ1*05:02 carrier	No	**0.015**	Ref	1
	Yes		3.922	1.311–11.734

Abbreviations: 95% CI, 95% confidence interval; AQ, areca‐quid chewing; OR, odds ratio. Bold numbers denote *p* value < 0.05.

### 3.3 Onset Age According to HLA‐HQB1*0502 Genotype in Validation Cohort

**FIGURE 2:**
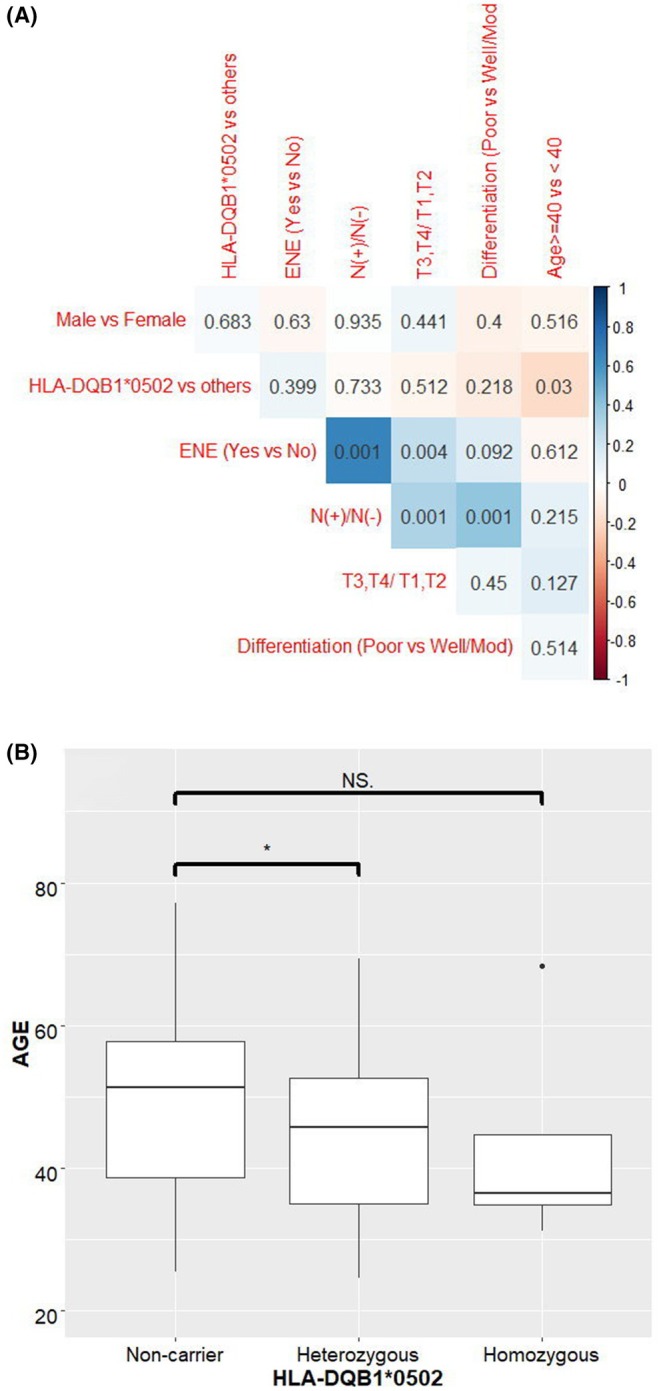
(A) Age distribution of 100 OSCC patients according to HLA‐DQB1*05:02 genotype. Patients carrying DQB1*05:02 had a younger age of OSCC onset. (B) Correlation plot between clinicopathological factors and HLA‐DQB1*05:02 genotype.

We analyzed the age distribution of 10 OSCC patients in the validation cohort using a histogram and determined the age difference according to the HLA‐DQB1*05:02 carrier status using an independent *t*‐test (Figure 2A). We found that patients carrying HLA‐DQB1*05:02 had a younger age of onset compared with non‐HLA‐DQB1*05:02 carriers (44.57 vs. 49.09 years; *p* = 0.051). Among homozygous HLA‐DQB1*05:02 carriers, the age of onset was 43.14 years (*p* = 0.536), with a trend toward a younger age of onset compared with heterozygous carriers and homozygous noncarriers (Figure 2B). The lack of a significant difference in onset age between homozygous carriers and noncarriers of HLA‐DQB1*05:02 can be attributed to the small number of patients (*n* = 4).

### 3.4 Correlation Between Clinicopathological Factors and HLA‐DQB1*05:02 Genotypes in Validation Cohort

We evaluated the correlations between HLA‐DQB1*05:02 genotypes and clinicopathological factors, such as tumor stage, age (< 40 vs. ≥ 40 years), lymph node metastasis, extra–nodal extension (ENE), and tumor differentiation (Figure 2B). The tumor stage was positively correlated with lymph node metastasis (*p* = 0.001) and ENE (*p* = 0.008). Furthermore, tumor cell differentiation was correlated with ENE (*p* = 0.034) and lymph node metastasis (*p* = 0.001). HLA‐DQB1*05:02 was correlated with a younger age of onset (*p* = 0.030). None of the clinicopathological factors (T stage, lymph node ENE, and nodal metastasis) were correlated with HLA‐DQB1*05:02.

Survival analysis by log‐rank test showed no significant associations between HLA‐DQB1*05:02 and disease‐free survival (log‐rank test: *p* = 0.068) or overall survival (log‐rank test: *p* = 0.416). The two HLA‐DQB1 loci were not significantly associated with patient survival.

## 4 Discussion

The association between HLA class I/II haplotype and tumorigenesis has been demonstrated for various tumors [15, 16, 17, 18]. Kubler et al. [19] demonstrated significantly higher occurrences of HLA haplotypes DRB1*03:01‐DQA1*0501‐DQB1*02:01 and DRB1*10:01‐DQA1*01:01‐DQB1*05:01 in patients with ovarian cancer. These findings suggest that HLA class II loci or certain haplotypes play a role in the development of ovarian cancer. Similarly, Koskinen et al. [20] and Reinders et al. [21] demonstrated associations between HLA DRB1, HLA DQB1, and HNC. Notably, the presence of the DQA1*01:02 allele has been linked to resistance against *Helicobacter pylori* ‐associated gastric atrophy and associated intestinal‐type gastric adenocarcinoma, whereas the absence of this allele represents a host genetic risk factor for these conditions [16]. The association of HLA‐A2 with nasopharyngeal carcinoma is limited to HLA‐A*02:07, which explains the observed associations between HLA‐A2 and nasopharyngeal carcinoma among Chinese [17].

HLA‐DQ and HLA‐DQB1 have rarely been reported to be associated with HNC [20, 21, 22, 23, 24, 25, 26]. Liu et al. [25] performed the largest multiethnic cohort study to date and found an association between HLA‐DQ‐DR and HNC. Tsai et al. [24] demonstrated no significant difference in the HLA‐DQA1* or HLA‐DQB1* allele frequency between OSCC patients and controls. The haplotype frequency of HLA*DQA1‐01:03‐DQB1*06:01 was significantly lower in OSCC patients than in controls (OR: 0.18, 95% CI: 0.054–0.583, corrected *p* = 0.02). Furthermore, HLA‐B*35 (OR = 0.31, *p* = 0.014) and HLA‐B*40 (OR = 2.9, *p* = 0.013) were significantly associated with OSCC and metastasis. Additionally, the HLA‐B*40‐DRB1*13 haplotype was more common in patients with OSCC (OR = 4.1, *p* = 0.016) [21]. Bau et al. found that HLA‐DQB1 was significantly different between OSCC and the normal population [26]. The specific loci and genotypes in HLA‐DQB1 were not further evaluated in the above study.

The HLA system, encoded on the short arm of chromosome 6, comprises two classes, HLA classes I and II, each characterized by distinct structures and functions [27]. As presented in Table 2, HLA‐DQB1 on chromosome 6 exhibited a high correlation with early‐onset OSCC. HLA‐DQB1, included in the HLA class II beta chain paralogs, is a heterodimer composed of an alpha chain (DQA) and a beta chain (DQB), both anchored in the membrane. DQA and DQB contain polymorphisms that determine the peptide‐binding specificities of the DQ molecule [28]. The presentation of appropriate peptides by MHC class II molecules is essential for immune function in the host [28]. MHC class II is loaded with extracellular proteins and is involved mainly in presenting extracellular pathogens. Oral microbiota such as *Porphyromonas gingivalis* , *Fusobacterium nucleatum* , and *Streptococcus* sp. have been implicated in OSCC carcinogenesis [29]. Poor dental hygiene along with certain environmental exposures, such as AQ consumption, alcohol consumption, and cigarette smoking, may interact synergistically with the HLA‐DQB1 polymorphism. The complex host responses to the microbiota can increase the risk of OSCC.

OSCC has a particularly high incidence in Taiwan and Southeast Asia due to exposure to AQ in this region. Complex gene–environment interactions increase the susceptibility of individuals with HLA‐DQB1*05:02 to OSCC in regions with high exposure to AQ. Further molecular and epidemiological studies are needed to confirm the associations of genetic loci with susceptibility to OSCC. The development of OSCC spans decades, progressing from premalignancy (such as leukoplakia and erythroplakia) to malignancy. The role of these HLA genetic markers in the premalignancy stage should be further investigated to determine its significance in the development of OSCC.

### 4.1 Limitation

This study was limited by the small OSCC case number in the GWAS analysis in the discovery cohort. Generally, the frequency of young age OSCC was low in all OSCC patients. In the future, more cases in the young age OSCC will be recruited for further analysis and validation.

## 5 Conclusion

This hospital‐based cohort study demonstrated a novel HLA locus, HLA‐DQB1*05:02, related to the increased risk of OSCC. Although this locus confers an increased risk of OSCC, environmental exposure could further determine the age of onset and subsite of the disease. In the general population, HLA‐DQB1*05:02 carriers should be particularly followed up regularly, whereas probands with OSCC and DQB1*05:02 carriers should be monitored for mucosal abnormalities in the head and neck region.

## Author Contributions

**Shiang‐Fu Huang:** conceptualization (equal), data curation (equal), funding acquisition (equal), investigation (equal), supervision (equal), visualization (equal), writing – original draft (equal), writing – review and editing (equal). **Huei‐Tzu Chien:** data curation (equal), investigation (equal), software (equal), writing – original draft (equal). **Chi‐Kuang Young:** data curation (equal), investigation (equal), resources (equal), software (equal), writing – review and editing (equal). **Yun‐Shien Lee:** data curation (equal), formal analysis (equal), investigation (equal), methodology (equal), software (equal), validation (equal), visualization (equal), writing – review and editing (equal). **Chun‐Ta Liao:** data curation (equal), investigation (equal), resources (equal), validation (equal). **Kai‐Lun Cho:** investigation (equal), methodology (equal), visualization (equal), writing – review and editing (equal). **Ching‐Han Chen:** data curation (equal), investigation (equal), methodology (equal), writing – review and editing (equal). **Angel Chao:** investigation (equal), validation (equal), visualization (equal), writing – review and editing (equal).

## Ethics Statement

This study was performed in compliance with the Declaration of Helsinki. The study was approved by the Institutional Review Board of Chang Gung Memorial Hospital (IRB no. 201800439B0) and written consent was obtained from all participants. Ethnically and geographically matched healthy controls from a biobank as a nationwide population study were approved by Taipei Medical University (TMU‐201805076).

## Conflicts of Interest

The authors declare no conflicts of interest.

## Data Availability

Data are available upon request to the corresponding author (Dr. Huang, S.F.).
